# Practices, barriers, and opportunities for dietitians‐nutrionists in critical care in Latin America: A cross‐sectional study

**DOI:** 10.1002/jpen.70074

**Published:** 2026-03-25

**Authors:** Mirta Crovetto, Solange Parra‐Soto, Samuel Durán‐Aguero, Karla Cordón‐Arrivillaga, Marisa Canicoba, Leslie Landaeta‐Díaz, Jhon Jairo Bejarano, Saby Mauricio, Edna J. Nava‐González, Valeria Carpio‐Arias, Sheila Cerezo, Sonia Ivankovich, Alfonsina Ortiz, Beatriz Nuñez‐Martinez, Eliana Romina Meza‐Miranda, Gertrudis M Adrianza de Baptista, Marisela Morales, Melissa Miranda‐Durán, Frank Carrera‐Gil

**Affiliations:** ^1^ Facultad de Salud y Ciencias Sociales Universidad de las Américas Chile; ^2^ Departamento de Nutrición y Salud Pública, Facultad de Ciencias de la Salud y de los Alimentos Universidad del Bío‐Bío Chillán Chile; ^3^ Escuela de Nutrición y Dietética, Facultad de Ciencias de la Rehabilitación y Calidad de Vida Universidad San Sebastián Santiago Chile; ^4^ Unidad de Investigación en Seguridad Alimentaria y Nutricional, Escuela de Nutrición, Facultad de Ciencias Químicas y Farmacia Universidad de San Carlos de Guatemala Guatemala City Guatemala; ^5^ Departamento de Alimentación y Dietética Hospital Nacional Profesor Alejandro Posadas, Provincia de Buenos Aires Argentina; ^6^ Núcleo de investigación en Nutrición y Ciencias alimentarias (NINCAL), Universidad de Las Américas Chile; ^7^ Departamento de Nutrición Humana, Facultad de Medicina Universidad Nacional de Colombia Bogotá Colombia; ^8^ Programa Académico Nutrición y Dietética, Universidad Privada Norbert Wiener Lima Peru; ^9^ Facultad de Salud Pública y Nutrición, Universidad Autónoma de Nuevo León México; ^10^ Grupo de Investigación en Alimentación y Nutrición Humana GIANH, Facultad de Salud Pública, Escuela Superior Politécnica de Chimborazo Chimborazo Ecuador; ^11^ Escuela de Nutrición, Universidad Interamericana de Panamá (UIP) Panamá City Panamá; ^12^ Asociación Costarricense de Dietistas y Nutricionistas San José Costa Rica; ^13^ Licenciatura en Nutrición, Facultad de Ciencias de la Salud, Universidad Católica del Uruguay Montevideo Uruguay; ^14^ Coordinación General de Investigación de la Universidad del Norte (Uninorte) Asunción Paraguay; ^15^ Centro Multidisciplinario de Investigaciones Tecnológicas, Universidad Nacional de Asunción San Lorenzo Paraguay; ^16^ Facultad de Medicina, Universidad Central de Venezuela. Universidad del Zulia Caracas Venezuela; ^17^ Instituto Salvadoreño de Bienestar Magisterial (ISBM), Centro de Hemodiálisis de El Salvador San Salvador El Salvador; ^18^ Posgrado de Facultad de Medicina, Enfermería, Nutrición y Tecnología Médica, Universidad Mayor de San Andrés La Paz Bolivia; ^19^ Departamento de Alimentación y Nutrición, Facultad de Ciencias de la Salud Pontificia Universidad Javeriana Seccional Cali Cali Colombia

**Keywords:** critical care, dietitian, intensive care unit, nutrition

## Abstract

**Background:**

Dietitians–nutritionists (DNs) play a key role in intensive care units (ICUs), yet their scope of practice varies across settings. This study aimed to describe the professional practices of DNs working in ICUs in Latin America and to identify factors associated with their level of professional activity.

**Methods:**

A cross‐sectional study was conducted using an online survey targeting DNs working in ICUs across 18 Latin American countries. The survey included two questionnaires: one assessing professional functions and responsibilities, and another evaluating practices, dynamics, and resources for nutritional care.

**Results:**

A total of 223 responses were obtained, mainly from Argentina, Chile, Colombia, Peru, and Mexico. Respondents reported a median of 10 years of professional experience (interquartile range: 6–16). Most worked in high‐complexity public hospitals (141 [63.2%]) and reported ≥40 h workweeks (144 [64.6%]); 137 (61.4%) held postgraduate degrees. Systematic participation in nutritional assessment, intervention, and monitoring was reported by 165 (74.0%), 183 (82.1%), and 168 (75.3%) respondents, respectively. Only 97 (43.5%) were authorized to prescribe nutritional therapy, with institutional policies identified as the main barrier. Academic involvement was limited, with 91 (41.7%) occasionally participating in teaching and 93 (42.1%) in research. The NRS‐2002 was the most commonly used screening tool; only 16 (7.2%) reported access to indirect calorimetry, and 120 (53.8%) assessed muscle mass, mainly by anthropometry

**Conclusions:**

DNs in Latin America play an active role in ICU nutritional care but face important limitations in clinical autonomy, access to diagnostic tools, and academic participation.

## INTRODUCTION

Strong evidence supports the link between nutritional status and clinical outcomes in hospitalized patients^1^, ^2^, ^3^, ^4^ as well as the essential role of nutritional care in reducing the consequences of disease‐related malnutrition.^5^ In particular, critical illness induces significant disruptions in nutrient metabolism^1^, resulting in substantial loss of lean body mass and impaired physiological function.^6^, ^7^ These alterations are associated with poorer clinical outcomes and a reduced quality of life.^8^, ^9^

Optimal nutritional therapy may contribute to improved clinical outcomes in intensive care units (ICUs)^10^, ^11^, ^12^, and clinical guidelines developed by international societies provide recommendations for nutritional assessment and therapy during critical illness.^13^, ^14^ However, their implementation in routine practice remains challenging due to variability in local resources, limited training, and organizational barriers.

In this context, trained clinical nutrition professionals play a central role in the provision of nutritional care and in addressing these limitations. In most Latin American countries, the legally recognized professional responsible for nutritional care is the nutritionist, typically holding a university degree in Nutrition or Nutrition and Dietetics. While the term dietitian is part of the official professional title in some countries (e.g., Colombia), both terms generally refer to the same regulated health professional with comparable training and scope of practice. Dietitians–Nutritionists (DNs) perform nutritional assessment, treatment planning, and ongoing monitoring.They contribute to the nutritional education of other healthcare professionals and promote standardized nutrition practices for critically ill patients.

Despite their importance, the practices of DNs in ICUs are not well documented, particularly in Latin America. Thus, the aim of this study was to describe the professional practices of DNs in ICUs, as well the challenges they face in providing nutritional care in Latin America. Additionally, it sought to evaluate the association between a Professional Activity Score (PAS) and the sociodemographic and professional characteristics of DNs. Understanding how professional activity varies according to these characteristics may help identify gaps in training, experience, or institutional support that could affect the quality of nutritional care in ICUs. This information is essential for guiding targeted strategies to strengthen the role of DNs in critical care settings across Latin America.

## MATERIAL AND METHODS

### Study design and participants

A cross‐sectional study using a convenience sample was conducted between March and May 2024 to explore the roles and work environments of DNs working in ICUs. Professionals from 18 countries—Argentina, Bolivia, Chile, Colombia, Costa Rica, Cuba, Ecuador, El Salvador, Guatemala, Honduras, México, Nicaragua, Panamá, Paraguay, Peru, República Dominicana, Uruguay, and Venezuela—were invited to participate. This study was not registered.

### Participant recruitment and eligibility criteria

Participant recruitment was coordinated by members of the Red Internacional de Nutricionistas‐Dietistas de América Latina (RINDAL), a collaborative network formed by the study co‐investigators from 13 Latin American countries. Each member acted as a national focal point, facilitating survey dissemination within their respective country.

A recruitment poster containing a brief study description and a Quick Response (QR) code linked to an online survey was designed and distributed digitally through social media platforms (including WhatsApp, Facebook, LinkedIn, and Instagram), institutional mailing lists, professional networks, national professional associations, universities, and clinical institutions. Reminder messages were disseminated periodically throughout the data collection period to enhance participation.

Eligible participants were DNs working in hospital settings with at least six months of experience in ICUs, regardless of employment in public or private institutions. Electronic informed consent was obtained prior to survey participation.

### Instruments and data collection

Data were collected using a single online survey administered in Spanish via Google Forms, which integrated two questionnaires designed to assess complementary aspects of professional practice among DNs working in ICUs.

**Figure 1:**
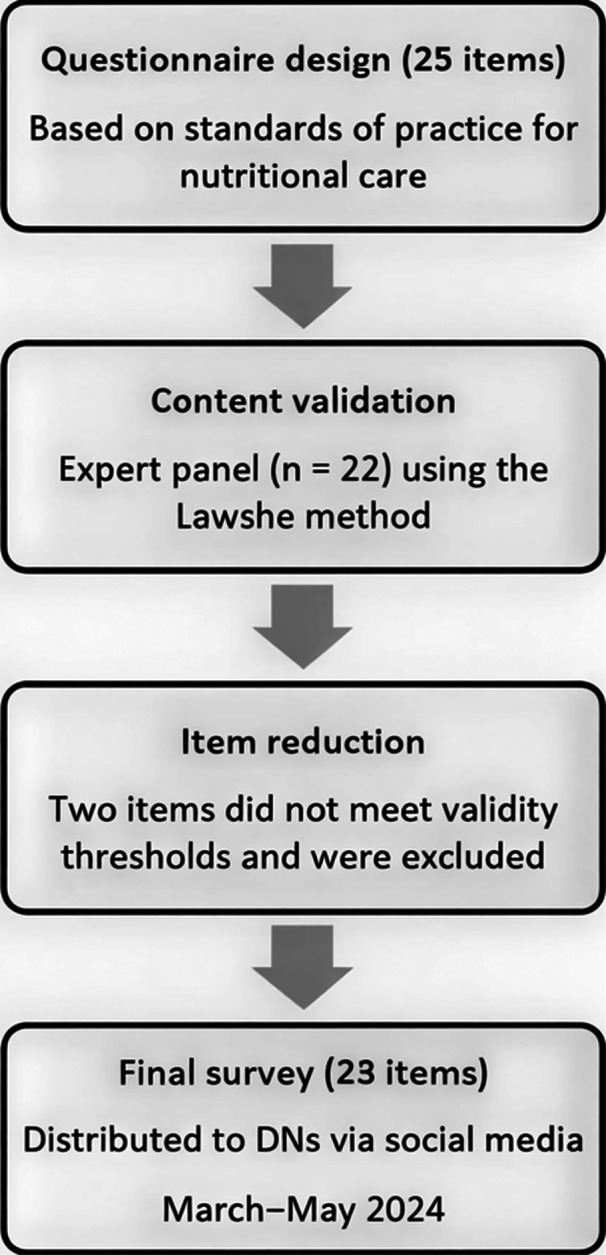
Flowchart of questionnaire development, validation, and distribution. DNs, dietians‐nutritionists.

The first questionnaire assessed practices, dynamics, and resources for nutritional care in ICUs and was specifically developed for this study. The initial version comprised 25 items and underwent a content validation process (Figure 1) conducted by a panel of 22 clinical nutrition experts. Content validity was evaluated using the Content Validity Index (CVI) and the Content Validity Ratio (CVR), following the method proposed by Lawshe.^15^ Items meeting predefined validity thresholds were retained, resulting in a final instrument of 23 items and an overall CVI of 0.87, indicating strong content validity. This questionnaire included closed‐ended questions aligned with international clinical nutrition guidelines and professional association documents. The validated instrument is provided in File S1.

The second questionnaire evaluated participation in professional functions and responsibilities using a previously validated instrument that measures the frequency of core clinical nutrition activities performed in hospital settings.^16^ For the purposes of this study, an adapted 17‐item version of the original 19‐item questionnaire was used. Based on this instrument, a PAS was constructed to quantify engagement in professional practice. Each item was scored as 1 point when always performed, 0.5 points when sometimes performed, and 0 points when never performed, yielding a total score ranging from 0 to 17, with higher scores indicating greater participation in professional activities. The activities included in the PAS correspond to the functions and responsibilities detailed in Table 2, and a glossary of key terms and definitions is provided in File S2.

### Data curation and statistical analysis

After data curation, items 4–9, 22, and 23 of the questionnaire validated in this study were excluded due to low response rates (<20%), as this could compromise the representativeness of the findings. Descriptive statistics were presented as numbers and percentages for categorical variables, and as medians with interquartile range (IQR) for continuous variables.

A multivariable linear regression analysis was conducted to assess the association between the PAS and sociodemographic and professional characteristics of DNs.The dependent variable was the PAS, which reflects the frequency of engagement in professional activities. The independent variables included sex (male vs. female), type of institution (private vs. public), years of professional experience (continuous), work schedule (full‐time vs. part‐time), level of hospital complexity (low, medium, high), and postgraduate education (yes vs. no). All variables were included simultaneously into a single multivariable linear regression model. No additional adjustments were made for potential confounding variables beyond those explicitly listed.

Model assumptions were evaluated prior to regression analyses through residual diagnostics, including visual inspection of residual histograms and normal probability (Q–Q) plots, assessment of homoscedasticity using residual plots, and evaluation of multicollinearity using variance inflation factors (VIF). All VIF values were below commonly accepted thresholds (mean VIF = 1.07), indicating no relevant multicollinearity.

Missing data were handled using a complete‐case analysis. Participants with missing values in the outcome variable or in any covariates included in the regression models were excluded.

All statistical analyses were performed using STATA version 18. A significance level of p < 0.05 with 95% confidence intervals was applied.

### Ethics

The research was conducted following the provisions of the Declaration of Helsinki and the project No. 007‐2022 which was approved by the Scientific Ethics Committee of the Universidad de Playa Ancha, Valparaíso, Chile.

## RESULTS

### Demographic, educational, and employment characteristics (Table 1)

**Table 1:** Demographic, educational, and employment characteristics.

	*n* = 223
**Sex,** ***n*** **(%)**	
Female	194 (87.0%)
Male	29 (13.0%)
**Experience as dietitians (years), Median (IQR)**	10 (6‐16)
**Country**	
Argentina	44 (19.7%)
Chile	36 (16.1%)
Colombia	32 (14.3%)
Perú	25 (11.2%)
México	24 (10.8%)
Ecuador	10 (4.5%)
Guatemala	10 (4.5%)
Others (countries with *n* < 10 participants)*	42 (18.8%)
**Institution type,** ***n*** **(%)**	
Public Hospital	140 (62.8%)
Private Hospital	60 (26.9%)
Public University Hospital	12 (5.3%)
Private University Hospital	11 (4.9%)
**Hospital complexity,** ***n*** **(%)**	
High	141 (63.2%)
Medium	69 (30.9%)
Low	13 (5.8%)
**Type of intensive care unit,** ***n*** **(%)**	
Adult	152 (70.7%)
Both (Adult and Pediatric)	45 (20.9%)
Pediatric	18 (8.4%)
Not informed	8 (3.7%)
**Work schedule,** ***n*** **(%)**	
Full‐time (40–45 h per week)	144 (64.6%)
Part‐time (20–36 h per week)	68 (30.5%)
Quarter‐time (10–12 h per week)	11 (4.9%)
**Postgraduate education in clinical nutrition,** ***n*** **(%)**	
Diploma	88 (39.5%)
Master's Degree	44 (19.7%)
Doctorate′s Degree	3 (1.3%)
Specialization	2 (0.9%)
No Postgraduate	86 (38.6%)

Abbreviation: IQR, Interquartile Range.

* Costa Rica (*n* = 8), Panamá (*n* = 8), Paraguay (*n* = 7), Uruguay (*n* = 7), El Salvador (*n* = 3), Bolivia (*n* = 2), Nicaragua (*n* = 2), Venezuela (*n* = 2), Cuba (*n* = 1), Honduras (*n* = 1), República Dominicana (*n* = 1).

A total of 223 responses were received from DNs across 18 Latin American countries. The number of participants per country ranged from 1 to 44, with the highest participation from Argentina (44 [19.7%]), Chile (36 [16.1%]), Colombia (32 [14.3%]), Peru (25 [11.2%]), and Mexico (24 [10.8%]). The median professional experience was 10 years (IQR: 6–16). Most respondents were women (194 [87.0%]) and were primarily employed in public, high‐complexity hospitals (141 [63.2%]). A similar proportion reported working a standard 40–45 h workweek (144 [64.6%]).

Regarding work settings, 152 (70.7%) worked exclusively in adult ICUs, 18 (8.4%) in pediatric ICUs, and 45 (20.9%) in both adult and pediatric ICUs; 8 (3.7%) did not specify the type of ICU. Most respondents held a postgraduate degree in clinical nutrition (137 [61.4%]), most commonly diplomas (88 [39.5%]), followed by master′s degrees (44 [19.7%]). A smaller proportion held doctoral degrees (3 [1.3%]) or specializations (2 [0.9%]).

### Functions and responsibilities (Table 2)

**Table 2:** Functions and responsibilities.

	Always	Sometimes	Never
**Nutritional assessment, intervention, and monitoring**			
Conduct Nutritional Assessment and Diagnosis in Patients Who Require It	165 (74.0%)	54 (24.2%)	4 (1.8%)
Perform Nutritional Intervention in Patients Who Require It	183 (82.1%)	38 (17.0%)	2 (0.9%)
Monitor Nutritional Progress and Outcomes	168 (75.3%)	51 (22.9%)	4 (1.8%)
Follow Clinical Guidelines for Nutritional Care	160 (71.7%)	52 (23.3%)	11 (4.9%)
Patients Undergo Nutritional Assessment	129 (57.8%)	86 (38.6%)	8 (3.6%)
Create Nutritional Care Plans for At‐Risk Patients	171 (76.7%)	46 (20.6%)	6 (2.7%)
Assess Nutritional Status and Identify Needs	174 (78.0%)	47 (21.1%)	2 (0.9%)
Offer Nutritional Counseling and Discharge Diet Plans	90 (40.4%)	106 (47.5%)	27 (12.1%)
Provide Nutrition Education to Patients and Families	100 (44.8%)	102 (45.7%)	21 (9.4%)
**Collaborative work, clinical documentation, teaching, and research**			
Work with Food Service to Evaluate Diet Quality	119 (53.4%)	77 (34.5%)	27 (12.1%)
Attend Clinical Sessions Focused on Patient Therapy	54 (24.2%)	110 (49.3%)	59 (26.5%)
Participate in Health Team and Dietitian Meetings	103 (46.2%)	102 (45.7%)	18 (8.1%)
Update and Maintain the Hospital Diet Manual	67 (30.0%)	84 (37.7%)	72 (32.3%)
Update the Discharge Nutrition Recommendations Manual	83 (37.2%)	97 (43.5%)	43 (19.3%)
Conduct administrative activities such as: controlling the quality of support	81 (36.3%)	93 (41.7%)	49 (22.0%)
Teach Undergraduate and Postgraduate Students	58 (26.0%)	93 (41.7%)	72 (32.3%)
Collaborate in Research Studies	23 (10.6%)	91 (42.1%)	102 (47.2%)

*Note*: Some items reflect related but distinct components of the nutritional care process (e.g., assessment, diagnosis, and identification of needs) and were analyzed separately as defined in the original validated questionnaire.^16^

#### Nutritional assessment, intervention, and monitoring

Systematic participation in nutritional assessment, intervention, and monitoring was reported by 165 (74.0%), 183 (82.1%), and 168 (75.3%) respondents, respectively. In contrast, 4 (1.8%) respondents reported never conducting nutritional assessments. Adherence to clinical practice guidelines for nutritional care was consistently reported by 160 (71.7%) participants.

Nutritional assessment of all ICU patients was always conducted by 129 (57.8%) respondents, sometimes by 86 (38.6%), and never by 8 (3.6%). Nutritional care plans for patients at risk of malnutrition were always developed by 171 (76.7%) respondents. Additionally, 174 (78.0%) reported always assessing nutritional status and calculating nutritional requirements.

Regarding documentation and education, 90 (40.4%) respondents always prepared detailed nutritional reports at discharge, and 100 (44.8%) consistently provided nutritional education to patients and their families.

#### Collaborative work, clinical documentation, teaching, and research

Collaboration with food services, including evaluation of meal composition, quality, and presentation, was always performed by 119 (53.4%) respondents, while 27 (12.1%) never engaged in this activity. Participation in multidisciplinary clinical meetings was always reported by 103 (46.2%) respondents, whereas 18 (8.1%) never participated.

For the development and updating of hospital diet manuals, 67 (30.0%) respondents reported always participating and 84 (37.7%) reported participating occasionally. Regarding discharge dietary recommendation manuals, 97 (43.5%) reported occasional participation, while 43 (19.3%) never participated.

Administrative and management activities, including quality control of nutritional support and staff supervision, were occasionally performed by 93 (41.7%) respondents, while 49 (22.0%) never engaged in these tasks. Involvement in undergraduate and postgraduate teaching was reported as occasional by 91 (41.7%) respondents, whereas 72 (32.3%) reported no teaching participation.

Participation in ICU research projects was reported as occasional by 91 (42.1%) respondents, while 102 (47.2%) reported no involvement. The main reasons for non‐participation included lack of time (84 [37.7%]), activities not being part of their professional role (77 [34.5%]), and assignment of these activities to other healthcare professionals (53 [23.8%]). Less frequently reported reasons included lack of interest (4 [1.8%]) and feeling unprepared (8 [3.6%]).

### Practices, dynamics, and resources for nutritional care (Table 3)

**Table 3:** Practices, dynamics, and resources for nutritional care.

**Nutritional screening methods (Multiple answers allowed)**	
Nutritional Risk Screening (NRS 2002)	123 (55.0%)
Subjective Global Assessment (SGA)	77 (34.5%)
Nutrition Risk in Critically Ill (NUTRIC)	67 (30.0%)
Malnutrition Universal Screening Tool (MUST)	30 (13.5%)
Malnutrition Screening Tool (MST)	28 (12.6%)
All patients are assessed without the need of screening process	22 (9.9%)
STRONGkids (Screening Tool for Risk of Nutritional Status and Growth)	22 (9.9%)
No screening conducted	21 (9.4%)
STAMP (Screening Tool for the Assessment of Malnutrition in Pediatrics)	4 (1.8%)
SNAQ (Short Nutritional Assessment Questionnaire)	1 (0.4%)
GLIM (Global Leadership Initiative on Malnutrition)	1 (0.4%)
MNA (Mini Nutritional Assessment)	1 (0.4%)
BIA (Bioelectrical Impedance Analysis)	1 (0.4%)
**Referral source for nutrition assessment**	
Physician	121 (54.3%)
Dietitian actively identifies patients	77 (34.5%)
Automated referral via screening system	7 (3.1%)
Other	18 (8.1%)
**Assessment delay after referral**	
Between 24 and 48 h	127 (57.0%)
Less than 24 h	83 (37.2%)
More than 48 h	13 (5.8%)
**Nutritional assessment documented in medical chart**	202 (90.6%)
**Nutrition‐related clinical documentation**	206 (92.4%)
**Evidence‐based nutrition documents**	194 (94.2%)
**Use of quality indicators in nutrition care**	129 (57.8%)
**Orders/Requests lab tests for nutrition care**	183 (82.1%)
**Reason for not ordering lab tests**	
No authorization to request tests	35 (87.5%)
Physician requests them/only physicians are allowed to request	2 (4.5%)
Patient already has necessary tests	1 (2.5%)
Lack of training to interpret results	1 (2.5%)
Use tests requested by medical team	1 (2.5%)
**Routine muscle mass/function tests**	**120 (53.8%)**
**Methods used to assess muscle status (Multiple answers allowed)**	
Arm or calf circumference	95 (42.6%)
Handgrip strength (dynamometer)	57 (25.6%)
BIA	22 (9.9%)
Muscle ultrasound	6 (2.7%)
Computed tomography	5 (2.2%)
Not performed	1 (0.4%)
**Energy requirement estimation methods (Multiple answers allowed)**	
Factorial method (weight × kcal)	136 (61.0%)
Predictive equations (e.g., Mifflin–St Jeor, Harris–Benedict)	110 (49.3%)
Clinical judgment	23 (10.3%)
Indirect calorimetry	16 (7.2%)
Other	6 (2.7%)
**Equipment for weighing bedridden patients**	89 (39.9%)
**Authorized to prescribe nutrition support**	
Both (Enteral and Parenteral)	97 (43.5%)
Only enteral	69 (30.9%)
Not authorized—I assist another professional	45 (20.2%)
Not authorized—Another professional prescribes without my assistance	8 (3.6%)
Only parenteral	4 (1.8%)
**Barriers to prescribing nutrition support (Multiple answers allowed)**	
Legal restrictions	39 (64.0%)
Institution policies	35 (66.0%)
Other	6 (10.0%)

For nutritional risk detection, 123 (55.0%) respondents reported using the Nutritional Risk Screening 2002 (NRS‐2002), while 77 (34.5%) used the Subjective Global Assessment (SGA). Additionally, 22 (9.9%) reported that all patients were assessed directly without a formal screening process. Most respondents indicated that physicians were the primary source of referrals for nutritional care (121 [54.3%]). The time between medical referral and nutritional assessment was reported as 24–48 h by 127 (57.0%) respondents. Proactive identification of patients requiring nutritional support was reported by 77 (34.5%), while screening integrated into the electronic medical record was reported by only 7 (3.1%).

Nutritional assessments were documented in the medical record by 202 (90.6%) respondents, and 129 (57.8%) reported using specific indicators to evaluate the quality of nutritional care. Laboratory tests to guide or assess nutritional therapy were ordered or recommended by 183 (82.1%) respondents. Among those who did not request laboratory tests (40 respondents), lack of authorization was cited as the main reason by 35 (87.5%).

Routine evaluation of muscle mass or function was reported by 120 (53.8%) respondents. The most frequently used methods were arm or calf circumference measurements (95 [42.6%]), followed by handgrip strength using a dynamometer (57 [25.6%]) and bioelectrical impedance analysis, BIA (22 [9.9%]).

Regarding the use of clinical documents and guidelines, 206 (92.4%) respondents reported consistent use, and 194 (94.2%) confirmed that these documents were evidence‐based. Equipment for weighing bedridden patients (beds with integrated scales or hoists) was available to 89 (39.9%) respondents. For energy requirement estimation, 136 (61.0%) used factorial methods, 110 (49.3%) used validated predictive equations, and 16 (7.2%) used indirect calorimetry.

Finally, authorization to prescribe both enteral and parenteral nutrition was reported by 97 (43.5%) respondents, while 69 (30.9%) were authorized to prescribe enteral nutrition only. Among respondents without prescribing authorization, institutional policies were cited as the main barrier by 35 (66.0%).

### Association between PAS and sociodemographic and professional characteristics (Table 4)

**Table 4:** Association Between Professional Activity Score (PAS) and sociodemographic and professional characteristics: Linear regression analysis.

Characteristics	Beta 95% CI	*P* value
Sex (Male)	1.10 (−2.01; 4.21)	0.486
Type of institution (Private)	1.32 (−0.93; 3.57)	0.249
Years experience	0.20 (0.06; 0.34)	0.004
Type of schedule (Half)	−1.00 (−3.88; 1.89)	0.495
Complexity hospital		
High	Ref.	
Low	−0.56 (−2.83; 1.71)	0.626
Medium	−2.40 (−6.91; 2.12)	0.297
Postgraduate (Yes)	0.73 (−1.55; 3.02)	0.527

There was a positive association between PAS and years of professional experience (*β* = 0.20, 95% CI: 0.06 to 0.34), indicating that each additional year of experience was associated with a 0.20‐point increase in the score. No statistically significant associations were found for sex (*β* = 1.10, 95% CI: −2.01 to 4.21), type of institution (*β* = 1.32, 95% CI: −0.93 to 3.57), part‐time work schedule (*β* = −1.00, 95% CI: −3.88 to 1.89), postgraduate education (*β* = 0.73, 95% CI: −1.55 to 3.02), or hospital complexity—whether low (*β* = −0.56, 95% CI: −2.83 to 1.71) or medium (*β* = −2.40, 95% CI: −6.91 to 2.12)—when compared to high‐complexity institutions.

## DISCUSSION

Critical illness represents a major risk factor for nutritional alterations, with important implications for patient survival and recovery. In this context, professionals with advanced training and experience in ICUs contribute substantially to the delivery of high‐quality nutritional care.

In our study, we observed a predominance of DNs with medium to high levels of experience (median of 10 years), most of whom held a postgraduate degree in clinical nutrition, highlighting a significant level of specialization. Professional experience is a key component of evidence‐based practice, as it facilitates decision‐making and promotes a deeper understanding of the importance and adaptation of evidence in clinical settings.^17^ Notably, we found that the DNs years of experience were significantly associated with greater participation in clinical activities, suggesting that a longer professional trajectory may influence the scope and execution of their responsibilities.

Moreover, the majority of participants reported a hospital workweek of 40 h or more. However, since it was not specified whether this workload was devoted exclusively to the ICU or also shared with other clinical areas, the actual time dedicated to ICU‐related activities remains uncertain. A French study among DNs found that increased time spent in the ICU increased the likelihood of consultation and consideration of their recommendations in therapeutic decisions.^18^ It has been observed that interventions delivered by DNs in the ICU can improve nutritional outcomes and contribute to better clinical results.^19^ Furthermore, involving a DN specialized in nutritional support in the ICU can enhance cost‐effectiveness and patient safety.^20^ This evidence underscores the need to integrate DNs into interdisciplinary ICU teams and to develop strategies that optimize their time in order to fully leverage their expertise.

Participants reported a high frequency of involvement in the assessment, intervention, and monitoring of patients requiring nutritional support. These activities represent key functions of DNs and demand the majority of their working time. However, it is important to note that other essential practices are performed only occasionally or not at all. These include providing nutritional education to patients and their families, participating in teaching and research activities, and attending clinical sessions where patient therapy is discussed.

While the role of the DN in the ICU as a clinician, educator, and researcher is vital to the multidisciplinary team^21^, several factors may hinder participation in these tasks. The main barrier reported by respondents was lack of time. This finding is consistent with a study conducted among Canadian DNs, which reported a desire for more frequent inter‐professional collaboration, but identified obstacles such as time constraints and limited awareness of their role.^22^ Similarly, a qualitative study in Saudi Arabia identified the limited knowledge among medical staff regarding the DNs role as major barrier^23^ —an issue also reported in a Latin American study.^24^

In contrast, a Swiss study interviewing ICU physicians, nurses, and speech therapists found that most regarded DNs as essential team members who enhanced nutritional care, with delegated prescribing perceived as valuable for improving management and balancing workload.^25^ These results underscore the urgent need for DNs to strengthen their presence in clinical meetings and research, particularly since their role in ICUs is still emerging.

Regarding nutritional education for patients and their families, only 44.8% of DNs reported always performing this activity. This represents a gap in care that warrants further investigation to explore DNs beliefs and attitudes toward nutritional counseling in the ICU. Frequent involvement in educational activities supports the integration of evidence into practice, increases exposure to scientific literature to answer patients′ questions, and maximizes the benefits of learning through teaching. However, it has been reported that DNs face challenges in identifying evidence‐based information related to counseling and communication.^26^

In relation to research, most of the DNs surveyed reported limited involvement in this area, likely due to educational factors in addition to lack of time.

A qualitative study found DNs often feel the need to improve their research skills, especially in statistics, as many graduates lack these competencies^27^ Another study reported that DNs typically see their role in research as supportive, with limited involvement in idea generation or study design.^26^ The American Dietetic Association has emphasized the importance of integrating research training into dietetics education programs and has proposed educational models that promote the development of these skills.^28^

Evidence supports including DNs as key members of ICU teams to provide high‐quality care.^29^ This collaboration is especially important in research, enhancing skills and rigor needed to generate scientific evidence.^30^

Regarding the work resources used by DNs for nutritional care, the NRS 2002 questionnaire was the most commonly employed for detecting nutritional risk. This instrument has demonstrated predictive validity in ICUs^31^ and is recommended for critical care by scientific nutrition societies.^14^ It is important to note that our questionnaire was specifically designed to capture nutritional risk screening practices. However, because respondents could freely add other tools, some mentioned SGA, BIA and the Global Leadership Initiative on Malnutrition (GLIM) criteria. These are nutritional assessment methods rather than screening instruments, and their appearance reflects open responses rather than the intended scope of the survey. The majority of respondents also reported using predictive equations to estimate resting energy expenditure, a practice commonly observed among DNs and documented in other studies.^32^ Although this method is widely used to determine caloric targets in the ICU, evidence has shown that it to be inaccurate, often leading to over‐ or underfeeding, which can be harmful.^33^, ^34^ Therefore, the development of affordable indirect calorimetry devices for routine use in ICUs is warranted.

Additionally, we observed that the assessment of muscle mass may be suboptimal, as it is routinely performed by only 54% of respondents, primarily using anthropometric measurements of the arm or calf, and to a lesser extent with technologies like BIA. The low routine measurement of muscle mass has also been reported by dietitians in Ireland, who cite lack of training and equipment availability for conducting these assessments.^35^ In critical illness, muscle wasting progresses rapidly as a result of systemic inflammation, increased catabolism, and prolonged immobility.^36^ This loss of muscle mass is linked to adverse clinical outcomes, including delayed recovery and prolonged convalescence.^37^ The GLIM consensus defines low muscle mass as a key criterion for diagnosing disease‐related malnutrition and recommends early and regular assessment in critically ill patients to enable timely detection.^38^ Moreover, a recent meta‐analysis found reduced muscle mass predicts survival in cancer cachexia better than low body mass index.^39^

Anthropometric measures are quick and accessible but less reliable with edema or inflammation. DNs working in ICU should be trained in BIA and muscle ultrasound, as these tools can detect acute muscle changes and guide nutritional interventions independently of body weight.^40^, ^41^ This is a crucial consideration, as only 39.9% of DNs in our study reported having adequate equipment to weigh immobile patients.

Finally, only 43.5% of respondents are authorized to prescribe enteral and parenteral nutrition, with institutional policies being the main barrier. Similar findings were reported in study across 132 Latin American hospitals, where dietitians prescribed nutritional therapy in 59% of cases^42^, and in Chilean hospitals, where their involvement in prescription was also limited.^43^ Additionally, surveys of DNs in the United States have yielded similar data, suggesting that this issue may be of universal concern.^44^ This is a key area for improvement, as it has been shown that prescriptions made by DNs positively impact the quality of nutritional support and patient safety, while also contributing to cost reduction in ICU care.^45^ For example, one audit reported fewer errors in parenteral nutrition prescriptions when made by DNs^46^, while another study demonstrated that granting DNs autonomy over enteral nutrition orders improved compliance and increased protein delivery in ICUs.^47^ Moreover, a recent study in 13 ICUs found that those with DNs offered more personalized nutrition, with gradual enteral feeding and better protein delivery. It also noted a low dietitian‐to‐bed ratio and stressed the need to enhance their role in critical care.^48^

These findings emphasize the critical role of DNs in the ICU, as optimal nutritional support is essential for preserving skeletal muscle in critically ill patients during early mobilization.^49^ They also highlight the need for further research into factors limiting dietitians′ authority to prescribe nutrition therapy in Latin American hospitals, particularly concerning country‐specific legal and educational frameworks. It is crucial that DNs take the lead in overcoming these barriers and maintaining competencies aligned with international standards.^50^ Enhancing their role will ensure safer access to nutritional therapies, helping to prevent malnutrition and reduce healthcare costs.

### Limitations and strengths

As far as we know, this is the first study that analyzes the practices of DNs in ICUs in Latin America with a significant sample size. The questions included in the survey contribute to a comprehensive approach to their practices and working conditions. Assessing professional performance through a PAS provides a baseline for comparison in future research. The findings offer evidence to better understand their contributions and challenges, make comparisons with other regions, and identify areas for improvement. This study has several limitations. Its cross‐sectional design precludes causal inference, and the uneven distribution of participants by country—including the absence of large countries such as Brazil—and their non‐random selection limit generalizability. In addition, allowing multiple responses per hospital may have led to duplicate reporting of barriers or experiences. The lack of information regarding exclusive time dedicated to ICU practice further limits the interpretation of professional engagement. Another limitation of our study was that items 4–9, 22, and 23 of the validated questionnaire used were excluded due to low response rates. Finally, reliance on self‐reports raises the possibility of selection and information bias.

## CONCLUSION

A high proportion of DNs in ICUs across Latin America have postgraduate training, extensive professional experience, and full work schedules dedicated to this service. The years of experience of DNs were associated with higher professional performance scores. Participation was greater in clinical activities and lower in patient education, research, and interdisciplinary clinical sessions. The lack of time was the main obstacle reported to participating in these tasks. Furthermore, less than half of DNs are involved in the prescription of enteral and parenteral nutrition, with institutional policies being the primary barrier reported. This situation suggests the need to establish policies that harmonize and regulate the role of DNs in these therapies. It also highlights the need for greater efforts to strengthen the participation and competencies of DNs in these areas, as well as further research with more representative samples of professionals from countries in this region.

## CLINICAL RELEVANCY STATEMENT

This study reveals challenges and practices of DNs in Latin American ICUs. Understanding these factors is key to optimizing nutritional therapy, improving patient outcomes, and guiding policies to strengthen DNs roles and autonomy in critical care.

## AUTHOR CONTRIBUTIONS

**Mirta Crovetto:** Writing—review and editing; supervision; conceptualization; investigation. **Solange Parra‐Soto:** Methodology; conceptualization; formal analysis; writing—original draft; writing—review and editing; data curation. **Samuel Durán‐Aguero:** Conceptualization; methodology; data curation; writing—original draft; writing—review and editing; formal analysis. **Karla Cordón‐Arrivillaga:** Methodology; writing—review and editing; investigation. **Marisa Canicoba:** Methodology; writing—review and editing; investigation. **Leslie Landaeta‐Díaz:** Methodology; writing—review and editing; investigation. **Jhon Jairo Bejarano:** Methodology; writing—review and editing; investigation. **Saby Mauricio:** Methodology; writing—review and editing; investigation. **Edna J. Nava‐González:** Methodology; writing—review and editing; investigation. **Valeria Carpio‐Arias:** Methodology; writing—review and editing; investigation. **Sheila Cerezo:** Methodology; writing—review and editing; investigation. **Sonia Ivankovich:** Methodology; writing—review and editing; investigation. **Alfonsina Ortiz:** Methodology; writing—review and editing; investigation. **Beatriz Nuñez‐Martinez:** Methodology; investigation; writing—review and editing. **Eliana Romina Meza‐Miranda:** Methodology; writing—review and editing; investigation. **Gertrudis M Adrianza de Baptista:** Writing—review and editing; investigation; methodology. **Marisela Morales:** Writing—original draft; writing—review and editing; methodology. **Melissa Miranda‐Durán:** Methodology; writing—original draft; writing—review and editing. **Frank Carrera‐Gil:** Methodology; data curation; formal analysis; writing—review and editing; writing—original draft; conceptualization. All authors are members of the Latin American Network for Research of Nutritionists‐Dietitians (RINDAL, by its initials in Spanish).

## CONFLICT OF INTEREST STATEMENT

None declared.

## Supporting information

Supplementary File 1.

Supplementary File 2.
