# Supplementary material for: Practices, barriers, and opportunities for dietitians‐nutrionists in critical care in Latin America: A cross‐sectional study
Source: JPEN J Parenter Enteral Nutr. 2026 Mar 25;50(4):582–92. doi: 10.1002/jpen.70074 (PMC13169267; doi:10.1002/jpen.70074)
Supplement: Supplementary file 1 [file JPEN-50-582-s002.docx]

**Supplementary File 1.**

**23-item validated questionnaire to assess practices, dynamics, and resources for nutritional care in intensive care units**

**1. Do you use documents such as procedure manuals, procedure guides, or protocols to perform your care activities as a dietitian-nutritionists?**

Yes / No

**2. If yes, were these documents developed following evidence-based nutrition criteria?**

Yes / No

**3. What tool is used for nutritional screening?**

Nutritional Risk Screening (NRS 2002)

Malnutrition Universal Screening Tool (MUST)

Nutritional screening is not performed

Other (specify)

**4. If screening is not performed, what is the main reason?***

It is not protocolized

Lack of time

Few dietitian-nutritionists available for the screening needs

All patients receive a nutritionist assessment without the need for screening

It is not a paid procedure

**5. Who usually performs nutritional screening?***

Dietitian-nutritionists

Nurse or Nursing Assistant

Self-reported by the patient

Pharmacist

Other (specify)

1. **How long does it usually take from hospital admission to nutritional screening?***

Less than 48 hours

More than 48 hours

**7. How many minutes does it take, on average, to perform nutritional screening?***

**8. Are the results of nutritional screening recorded in the patient's medical record?***

Yes / No

**9. What percentage of patients identified in nutritional screening as malnourished or at nutritional risk receive a nutritional assessment?***

Less than 25%

Between 25% and 50%

Between 50% and 75%

More than 75%

**10. Who usually refers patients to the nutritionist for a nutritional assessment?**

Doctor

Nurse

Electronic medical record system (automated referral, no intermediary)

No one, the dietitian-nutritionists actively searches in the inpatient units

Other (please specify)

**11. How long does it take from the time the patient is referred until the nutritional assessment is performed?**

Less than 24 hours

Between 24 and 48 hours

More than 48 hours

**12. Is the nutritional assessment recorded in the patient's medical record?**

Yes / No

**13. Do you use specific indicators to assess the quality of nutritional care?**

Yes / No

**14. Do you order or recommend laboratory tests to guide or evaluate the effectiveness of nutritional treatment?**

Yes / No

**15. If you do not order or recommend laboratory tests, what is the primary reason?**

I am not authorized.

They are not necessary.

I don't know how to interpret them.

There is no budget.

Other (please specify)

**16. Do you perform routine tests to assess muscle mass or function?**

Yes / No

**17. What is the common technique for studying muscle mass or function?**

Upper arm or calf circumference measurement.

Grip strength with a dynamometer.

Computed tomography.

Dual-energy X-ray absorptiometry (DEXA). Muscle ultrasound.

Bioimpedance.

Other (please specify)

**18. Do you have a bed or hoist for weighing bedridden patients?**

Yes / No

**19. What method do you use to calculate the energy needs of patients?**

Validated predictive equations (e.g., Mifflin St. John's, Harris Benedict, or other)

Factorial method (body weight multiplied by kilocalories)

Indirect calorimetry Clinical judgment

Other (please specify)

**20. Are you authorized to prescribe enteral and parenteral nutrition at your institution?**

Yes, both enteral and parenteral nutrition. Enteral nutrition only.

Parenteral nutrition only.

Neither. The prescription is written by another professional WITH my help.

Neither. The prescription is written by another professional WITHOUT my help.

**21. If nutritional support is not prescribed, what is the main reason that prevents this?**

Lack of adequate training

Institutional policies

Other (please specify)

1. **What is your monthly salary in your country’s currency? (Consider a full-time job or its equivalent of approximately 40 hours per week.)**

**23. If you have a specialty in clinical nutrition, is it recognized by**

Dietitian-Nutritionists' Association

Ministry of Education

Ministry of Health or equivalent,

Don't know

Other (please specify)
