# Supplementary material for: Practices, barriers, and opportunities for dietitians‐nutrionists in critical care in Latin America: A cross‐sectional study
Source: JPEN J Parenter Enteral Nutr. 2026 Mar 25;50(4):582–92. doi: 10.1002/jpen.70074 (PMC13169267; doi:10.1002/jpen.70074)
Supplement: Supplementary file 2 [file JPEN-50-582-s001.docx]

**Supplementary File 2.**

**Glossary of key terms and definitions of functions and responsibilities**

**Hospital Complexity:** The level of hospital complexity is determined, among other factors, by the type of financing and the hospitals purpose, which is classified according to standards and regulations that consider the number of beds, geographic distribution, medical specialties, and available technologies. Hospitals are classified as high, medium, or low complexity according to their patient care capacity. These criteria are based on the following: a) Function in the healthcare network, considering the different levels of network complexity; b) Support services for diagnosis and therapy, response capacity, availability of human resources and equipment, working hours, and procedures performed in the hospital; and c) Staff training level (1).

**Nutritional Intervention:** A planned and intentional process that seeks to improve nutritional status or nutrition-related health outcomes. It involves designing and implementing specific actions based on an individualized nutritional diagnosis, which may include everything from dietary changes, supplements, education, and counseling to adjustments in the healthcare environment. In clinical settings, the intervention may encompass multiple components: targeted nutrient provision (oral nutrition, supplements, enteral nutrition), nutritional education and counseling, and modifications to logistics or services, such as improving the dining environment or coordinating with other healthcare professionals (2).

**Hospital Diet Manual:** A technical and regulatory document that compiles, organizes, and describes the different types of diets used in healthcare institutions. Its purpose is to standardize medical prescriptions and the preparation of diets according to the nutritional needs of patients, considering their clinical conditions, physiological status, and therapeutic objectives. This manual establishes the classification, indications, nutritional characteristics, preparation, and distribution of each diet, facilitating collaborative work between physicians, nutritionists, and hospital kitchen staff. It is also a fundamental tool for ensuring food safety, the quality of nutritional care, and the continuity of care for hospitalized patients (3).

**Nutritional assessment:** A systematic process aimed at identifying and assessing the nutritional status of a person or population group. It is performed through the collection, analysis, and interpretation of clinical,anthropometric, dietary, biochemical, and functional data, with the goal of diagnosing malnutrition, excess weight, or other eating-related disorders and guiding appropriate nutritional interventions. It is considered an essential component of healthcare and hospital care, as it allows for the establishment of individualized nutritional care plans, monitoring progress, and preventing associated complications (4).

**Nutritional follow-up:** This is the continuous and systematic process by which the evolution of a patients nutritional status is monitored after a dietary or therapeutic intervention. It involves the periodic collection of clinical, anthropometric, dietary, and biochemical data to evaluate the effectiveness of the intervention, ensure adherence to the nutritional plan, prevent complications, and make necessary adjustments. This follow-up is an integral part of the Nutritional Care Process (NCP) and ensures continuity of care in hospital, outpatient, and community settings (5).

**Perform nutritional assessment and diagnosis in patients who require it: T**he professional responsibility of conducting a comprehensive nutritional assessment and establishing a formal nutritional diagnosis as part of the Nutritional Care Process (NCP), typically triggered by clinical indication or referral(6).

**Patients undergo a nutritional assessment:** The implementation or coverage of nutritional assessment at the patient level, indicating whether nutritional assessment is systematically applied to patients as part of routine care, regardless of who initiates it (7).

**Assess nutritional status and identify need:** The analytical step of interpreting assessment data to identify nutritional requirements and priorities, which may inform care planning even in the absence of a formal diagnosis.

**References**

1. Crovetto M, Durán-Aguero S, Parra-Soto S, et al. What is happening with the clinical nutritionist? Realities and challenges. Clin Nutr ESPEN. 2024;60:41-47. doi:10.1016/j.clnesp.2024.01.006

1. Wong A, Huang Y, Banks MD, Sowa PM, Bauer JD. A Conceptual Study on Characterizing the Complexity of Nutritional Interventions for Malnourished Older Adults in Hospital Settings: An Umbrella Review Approach. Healthcare (Basel). 2024;12(7):765. doi:10.3390/healthcare12070765
2. Ministry of Public Health and Social Welfare. Hospital Diet Manual [Internet]. Asunción: MSPBS; 2021 [cited 2025 Aug 19]. Available at: <https://www.mspbs.gov.py>
3. Cederholm T, Jensen GL, Correia MITD, Gonzalez MC, Fukushima R, Higashiguchi T, et al. GLIM criteria for the diagnosis of malnutrition – A consensus report from the global clinical nutrition community. Clin Nutr. 2019;38(1):1-9. doi:10.1016/j.clnu.2018.08.002.
4. Barazzoni R, Deutz NEP, Biolo G, Cederholm T, Cuerda C, Delzenne NM, et al. Monitoring nutrition in hospital patients: a position paper of the European Society for Clinical Nutrition and Metabolism (ESPEN). Clin Nutr. 2021;40(12):5082-96. doi:10.1016/j.clnu.2021.09.017.
5. Cederholm T, Jensen GL, Correia MITD, Gonzalez MC, Fukushima R, Higashiguchi T, et al. GLIM criteria for the diagnosis of malnutrition – Update and validation in clinical practice. Clin Nutr. 2022;41(6):1355-64. doi:10.1016/j.clnu.2022.02.004.
6. Mendes R, Amaral TF, Borges N. Nutritional assessment and monitoring in clinical practice: current perspectives. Clin Nutr ESPEN. 2022;48:1-8. doi:10.1016/j.clnesp.2022.01.007.
